# Changes in cardiac arrest patients’ temperature management after the publication of 2015 AHA guidelines for resuscitation in China

**DOI:** 10.1038/s41598-017-16044-7

**Published:** 2017-11-22

**Authors:** Lanfang Du, Baolan Ge, Qingbian Ma, Jianzhong Yang, Fengying Chen, Yuhong Mi, Huadong Zhu, Cong Wang, Yan Li, Hongbo Zhang, Rongjia Yang, Jian Guan, Yixiong Zhang, Guiyun Jin, Haiyan Zhu, Yan Xiong, Guoxing Wang, Zhengzhong Zhu, Haiyan Zhang, Yun Zhang, Jihong Zhu, Jie Li, Chao Lan, Hui Xiong

**Affiliations:** 1 Department of Emergency Medicine, The Peking University Third Hospital, No. 49, North Garden Rd., Haidian District, Beijing, 100191 China; 2 Department of Emergency Medicine, The First Affiliated Hospital of Xinjiang Medical University, No. 137, Liyushan South Rd., Wulumiqi, Xinjiang 830054 China; 3 Department of Emergency Medicine, The Affiliated Hospital of Innor Mongolia Medical University, No. 1, Tongdao North Rd., Huhehaote, Innor Mongolia, 010050 China; 4 Department of Emergency Medicine, Beijing Anzhen Hospital, 2 Anzhen Rd., Chaoyang District, Beijing, 100029 China; 5 Department of Emergency Medicine, Peking Union Medical College Hospital, No. 1, Shuaifuyuan Wangfujing Dongcheng District, Beijing, 100730 China; 6 Department of Emergency Medicine, Beijing Jishuitan Hospital, No. 31, Xinjiekou East Xicheng District, Beijing, 100035 China; 7 Department of Emergency Medicine, The Second Affiliated Hospital of Shanxi Medical University, No. 382, Wuyi Rd., Taiyuan, Shanxi 030001 China; 8 Department of Emergency Medicine, China Japan friendship hospital, No. 2, Yinghua East Rd., Chaoyang District, Beijing, 100029 China; 9 Department of Emergency Medicine, Gansu Provincial Hospital, No. 204, Donggang West Rd., Lanzhou, Gansu 730000 China; 10 Department of Emergency Medicine, The First Hospital of Tsinghua University, No. 6, Jiuxianqiao Yijiefang, Chaoyang District, Beijing, 100016 China; 11 Department of Emergency Medicine, Hunan Provincial People’s Hospital, No. 61, Jiefang West Rd., Changsha, Hunan 410005 China; 12 Department of Emergency Medicine, The Affiliated Hospital of Hainan Medical University, No. 31, Longhua Rd., Haikou, Hainan 570102 China; 13 Department of Emergency Medicine, The General Hospital of People’s Liberation Army, No. 28, Fuxing Rd., Beijing, 100853 China; 14 Department of Emergency Medicine, The First Affiliated Hospital of Sun Yat-sen University, No. 58, Zhongshan Second Rd., Guangzhou, Guangdong, 510080 China; 15 Department of Emergency Medicine, Beijing Friendship Hospital, No. 95, Yongan Rd., Xicheng District, 100050 China; 16 Department of Emergency Medicine, Beijing University Shougang Hospital, No. 9, Jinyuanzhuang Rd., Shijingshan District, Beijing, 100144 China; 17 Department of Emergency Medicine, The Hospital of Shunyi District Beijing, No. 3, Guangming South Street, Shunyi District, Beijing, 101300 China; 18 Department of Emergency Medicine, Beijing Tongren Hospital, No. 1, Dongjiaominxiang, Dongcheng District, Beijing, 100730 China; 19 Department of Emergency Medicine, Peking University People’s Hospital, No. 11, Xizhimen South Street, Xicheng District, Beijing, 100044 China; 20 Department of Emergency Medicine, Beijing Fuxing Hospital, No. 20, Fuxingmenwai Street, Xicheng District, Beijing, 100038 China; 21 Department of Emergency Medicine, The First Affiliated Hospital of Zhengzhou University, No. 1, Jianshe East Rd., Zhengzhou, Henan 450052 China; 22 Department of Emergency Medicine, Peking University First Hospital, No. 8, Xishiku Street, Xicheng District, Beijing, 100034 China

## Abstract

A survey was performed to assess the current management of targeted temperature management (TTM) in patients following cardiac arrest (CA) and whether healthcare providers will change target temperature after publication of 2015 American Heart Association guidelines for resuscitation in China. 52 hospitals were selected from whole of China between August to November 2016. All healthcare providers in EMs and/or ICUs of selected hospitals participated in the study. 1952 respondents fulfilled the survey (86.8%). TTM in CA patients was declared by 14.5% of physicians and 6.7% of the nurses. Only 4 of 64 departments, 7.8% of physicians and 5.7% of the nurses had implemented TH for CA patients. Since the publication of 2015 AHA guidelines, 33.6% of respondents declared no modification of target temperature, whereas 51.5% declared a target temperature’s change in future practice. Respondents were more likely to choose 35∼36 °C-TTM (54.7%) after guidelines publication, as compared to that before guidelines publication they preferred 32∼34 °C-TTM (54.0%). TTM for CA patients was still in the early stage in China. Publication of 2015 resuscitation guidelines did have impact on choice of target temperature among healthcare providers. They preferred 35∼36 °C-TTM after guidelines publication.

## Introduction

There were more than 500 thousand cardiac arrests (CA) each year in China. Even in Beijing-the capital of China-where the medical technology was well developed, the survival to hospital discharge and the percentage of patients with good neurological outcome (usually defined as cerebral performance category 1 and 2) was only 1.3% and 1.0% following out-of-hospital CA^1^, the survival to hospital discharge and the percentage of patients with good neurological outcome was9.1% and 6.4% following in-hospital CA^2^.

Targeted temperature management (TTM) is one of few beneficial interventions to improve outcomes of CA patients, it has been recommended as a standard therapy for comatose patients after return of spontaneous circulation (ROSC)^3,4^. It has been already more than one decade since the two landmark studies of therapeutic hypothermia (TH) after CA have been published^5,6^. Since then, the widespread implementation of TH seemed to occur^7–9^. But in China it was not until 2012 that TH began to be used for CA patients.

TH was recommended that CA patients should be cooled to 32 °C to 34 °C for 12 to 24 hours in 2010 American Heart Association (AHA) Guidelines for cardiopulmonary resuscitation (CPR)^10^. But with the new evidence, they found that all TTM procedures (e.g. 36 °C-TTM and 33 °C-TTM) are similarly beneficial when compared with non-TTM regimen^5,6,11–13^. Especially the large well-conducted randomized TTM trial revealed that no main outcome differences were observed between the two evaluated levels of TTM: 33 °C versus 36 °C^11^. So selecting and maintaining a constant temperature between 32 °C and 36 °C during TTM was recommended in 2015 AHA guidelines^3^.

The awareness and application of TTM in CA patients and whether healthcare providers will change target temperature after publication of 2015 AHA guidelines has never been assessed in China. We conducted a nationwide survey to determine the current awareness and management of TTM for CA patients in emergency departments (EDs) and ICUs.

## Methods

### Study design and data collection

**Figure 1:**
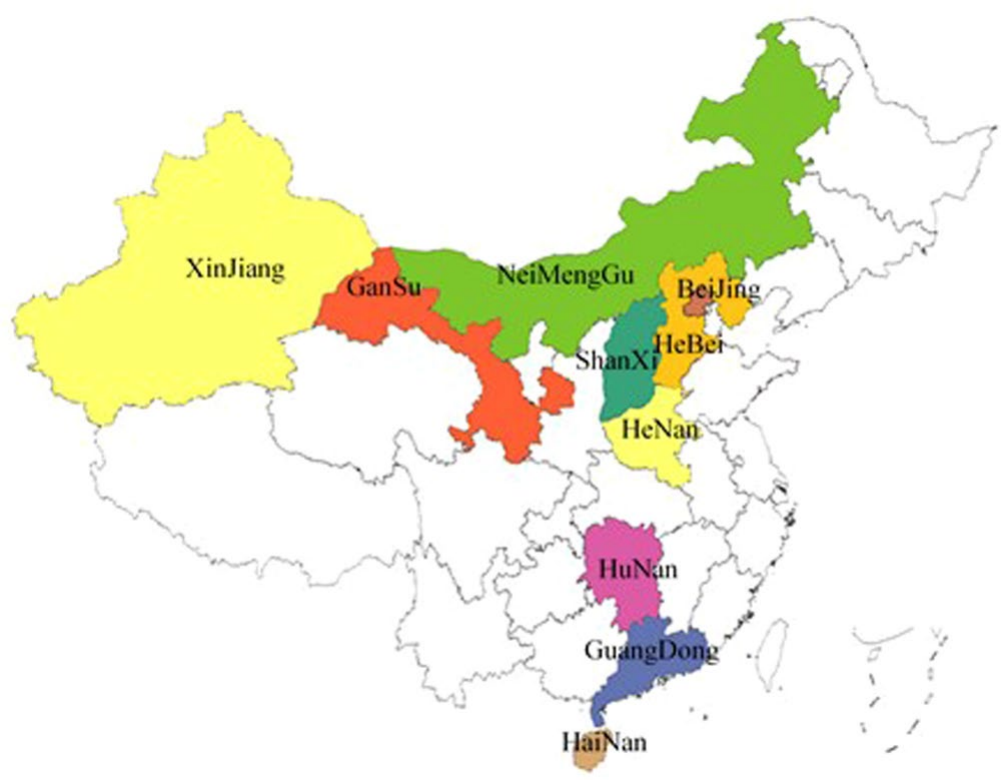
Geographic distribution of surveyed provinces http://echarts.baidu.com/ and the version URL is http://gallery.echartsjs.com/preview.html?c=xr1n8e1eO-.

This was a cross-sectional multicenter study. Multistage convenience sampling was used. First of all 10 representative provinces were selected from the east, south, west, north and middle of China. The 10 provinces were well geographically distributed as shown in Fig. 1 (5 provinces were in the relatively developed east and south of China, 3 provinces were in the relatively underdeveloped west and north of China and 2 provinces were in middle of China). Then we chose at least one affiliated hospital of a well-known medical university (representing hospitals with relatively advanced medical technology)and hospitals with varying numbers in second- and third-tier cities (representing hospitals with relatively underdeveloped medical technology) in select province based on the number and level of the province’s hospitals. Among those hospitals, we were more likely to choose hospitals which have a good cooperative relationship with us in order to get a high response rate and high quality control. Finally 52 hospitals were included, of which 47 are Level III and 5 are Level II hospitals. Then we conducted a questionnaire survey for all healthcare providers in the EDs and/or ICUs of selected hospitals, in an attempt to target providers likely to care for cardiac arrest patients. Informed consent was obtained from all participants.

The questionnaire was developed by a senior emergency physician experienced in CA and TTM and discussed 3 times by an emergency expert team working on rephrasing and improvement. Briefly, the questionnaire includes three parts: (1) Characteristics of the ICU or ED and the respondent; (2) General management of CA patients; (3) The awareness and practice of TTM in CA patients. The study group tested both test-retest reliability and split-half reliability of the questionnaire. Before the questionnaire was send to the respondents, test-retest reliability was analyzed. 105 healthcare providers, including 29 physicians and 76 nurses with reasonable distribution in different age and titles, were selected to fulfill the questionnaire two times in an interval of two weeks. There were no significant differences in the results of the repeated surveys and the Pearson correlation coefficient was 0.80 indicating a high quality in test-retest reliability. After data was collected from all respondents, split-half reliability was analyzed and the Guttman split-half coefficient of the questionnaire was 0.95 indicating a high quality in split-half reliability. The questionnaires were sent to the heads from August to November2016. Data collection ended in February 2017. All protocols were approved by Peking university third hospital. All methods were performed in accordance with the relevant guidelines and regulations.

### Statistical methods

The data were analyzed by SPSS 20.0. Quantitative variables were expressed as mean (standard deviation) when following a Gaussian distribution or median (inter quartile range 25∼75%) otherwise. Qualitative variables were expressed as frequencies. Data was tested for normality using the Shapiro-Wilk Normality Test.

## Results

A total of 2250 questionnaires were sent, 1952 respondents of 64 EDs or ICUs fulfilled the survey (86.8%). General characteristics of respondents and departments are described in Table 1.Only 4 of 64 departments (6.3%) had implemented TH for CA patients.3 departments performed ≤5 cases per year and one departments performed 5∼10 cases per year. General management of CA patients was shown in Table 2.

**Table 1:** Characteristics of respondents (number of respondents: n) and departments (number of departments: N).

Type of departments (N = 64)	
EDs	49(76.6)
ICUs	15(23.4)
Type of respondents (n = 1883)	
Doctors	905(48.1)
Nurses	978(51.9)
Title of doctor (n = 866)	
Resident doctor	421(48.6)
Attending doctor	301(34.8)
Associate chief physician	104(12.0)
Chief physician	40(4.6)
Title of nurse (n = 826)	
Nurse	421(51.0)
Nurse-in-charge	301(36.4)
Associate chief nurse	104(12.6)
Characteristics of departments (N = 64)	
Beds, mean ± SD	36 ± 19
Doctors, media (Q_1_,Q_3_)	14(7,26)
Nurses, media (Q_1_,Q_3_)	20(14,54)
Activity during one year period prior to the present study	
Admissions for CA	
<100	8(12.5)
100–300	18(28.1)
>300	38(59.4)
TH implementation for CA	
none	60(93.8)
<5	3(4.7)
5–10	1(1.6)
>10	0(0)

Results are expressed as n (%) unless specified otherwise. EDs: Emergency department; ICUs: Intensive care unit; CA: Cardiac arrest; TH: Therapeutic hypothermia.

**Table 2:** General management of CA patients (number of respondents: n).

Activities of respondents: admissions for CA during one-year period prior to the present study (n = 1891)	
<10	410(21.7)
10–30	528(27.9)
30–50	288(15.2)
>50	665(35.2)
Use of a written CA procedure (n = 1925)	911(47.3)
Performing coronary angiogram after ROSC (n = 1900)	
Always	158(8.3)
Frequently	380(20.0)
Sometimes	749(39.4)
Never	145(7.6)
Do not know	468(24.6)
The hospital has coronary intervention team (n = 1924)	1651(85.8)
With a 24 h availability (n = 1570)	1322(84.2)
Performing a brain CT or MRI scan after ROSC (n = 1916)	
Always	212(11.1)
Frequently	531(27.7)
Sometimes	701(36.6)
Never	64(3.3)
Do not know	408(21.3)
Target arterial blood pressure after ROSC	
SBP(n = 1029)	
<90 mmHg	131(12.7)
=90 mmHg	73(7.1)
>90 mmHg	814(79.1)
Others	11(1.1)
DBP(n = 904)	
>65 mmHg	673(74.4)
>70 mmHg	199(22.0)
Others	32(3.5)
MBP(n = 952)	
>60 mmHg	452(47.5)
>65 mmHg	484(50.8)
Others	16(1.7)
Use of cardiac-assisted devices in patients with hemodynamic instability after ROSC (n = 1919)	93(4.8)
If used, what kind of cardiac-assisted devices (n = 92)	
ECMO	36(39.1)
IABP	68(73.9)
Performing EEG after ROSC (n = 1870)	
Always	190(10.2)
Frequently	191(10.2)
Sometimes	615(32.9)
Never	517(27.6)
Do not know	357(19.1)

Results are expressed as n (%); CT: computerized-tomography; MRI: Magnetic Resonance Imaging; CA: Cardiac arrest; ROSC: return of spontaneous circulation; SBP: systolic arterial blood pressure; DBP: diastolic arterial blood pressure; MBP: mean arterial blood pressure; ECMO: extracorporeal membrane oxygenation; IABP: intra-aortic balloon pump; EEG: Electroencephalogram.

### The awareness of TTM in comatose adult patients with CA after ROSC

The awareness of TTM in comatose CA patients was described in Table 3. The ratio of respondents who did not know the concept of TTM recommended by guidelines was 77.1% in physicians and 86.2% in nurses, and that who did not know the concept of TH was 84.9% in physicians and 86.6% in nurses. 57.6% of physicians and 37.3% of nurses thought that intravascular cooling was better in temperature control and improvement of CA patients’ outcomes than that of surface cooling. But surface cooling was considered to be safer as reported by about 1/3 respondents. Considering the two target temperature levels of TTM (33 °C versus 36 °C), about 1/3 respondents believed that 33 °C-TTM was more beneficial in improving survival and neurologic outcomes of CA patients, but 44.0% of physicians and 31.8% of nurses thought that 36 °C-TTM had more advantages in security.

**Table 3:** The awareness of TTM in comatose adult patients with CA after ROSC (number of respondents: n).

	Physicians	Nurses
Kwon the concept of TTM	189/827(22.9)	128/927(13.8)
Kwon the concept of TH	134/888(15.1)	127/950(13.4)
Believe that TH can improve survival	677/873(77.5)	575/920(62.5)
Believe that TH can improve neurologic outcomes	721/871(82.8)	609/925(65.8)
Awareness of that active rewarming should be used	602/878(68.6)	510/931(54.8)
What do you think is the rewarming rate if active rewarming is used		
0.1–0.2 °C/h	217/594(36.5)	193/506(38.1)
0.3–0.53 °C/h	268/594(45.1)	199/506(39.3)
>0.5 °C/h	10/594(1.7)	41/506(8.1)
Do not know	99/594(16.7)	73/506(14.4)
Attitude to cooling methods: surface vs intravascular hypothermia		
Temperature control		
Surface cooling is better	105/884(11.9)	133/941(14.1)
Intravascular cooling is better	509/884(57.6)	351/941(37.3)
No bias	76/884(8.6)	125/941(13.3)
Do not know	194/884(21.9)	332/941(35.3)
Improvement of survival and neurologic outcomes		
Surface cooling is better	72/878(8.2)	100/933(10.7)
Intravascular cooling is better	482/878(55.4)	328/933(35.2)
No bias	107/878(12.2)	142/933(15.2)
Do not know	878/878(24.3)	363/933(38.9)
Security		
Surface cooling is better	286/875(32.7)	286/928(30.8)
Intravascular cooling is better	271/875(31.0)	218/928(23.5)
No bias	83/875(9.5)	95/928(10.2)
Do not know	235/875(26.9)	329/928(35.5)
Attitude to target temperature: 33 °C vs 36 °C		
Improvement of survival		
33 °C was better	321/873(36.8)	273/930(29.4)
36 °C was better	112/873(12.8)	184/930(19.8)
No bias	165/873(18.9)	116/930(12.5)
Do not know	275/873(31.5)	357/930(38.4)
Improvement of neurologic outcomes		
33 °C was better	364/871(41.8)	302/931(32.4)
36 °C was better	96/871(11.0)	171/931(18.4)
No bias	136/871(15.6)	89/931(9.6)
Do not know	275/871(31.6)	369/931(39.6)
Security		
33 °C was better	128/828(15.5)	169/922(18.3)
36 °C was better	364/828(44.0)	293/922(31.8)
No bias	121/828(14.6)	110/922(11.9)
Do not know	215/828(26.0)	350/922(38.0)

Results are expressed as n (%); TH: Therapeutic hypothermia.

### The practice of TTM in comatose adult patients with CA after ROSC

**Figure 2:**
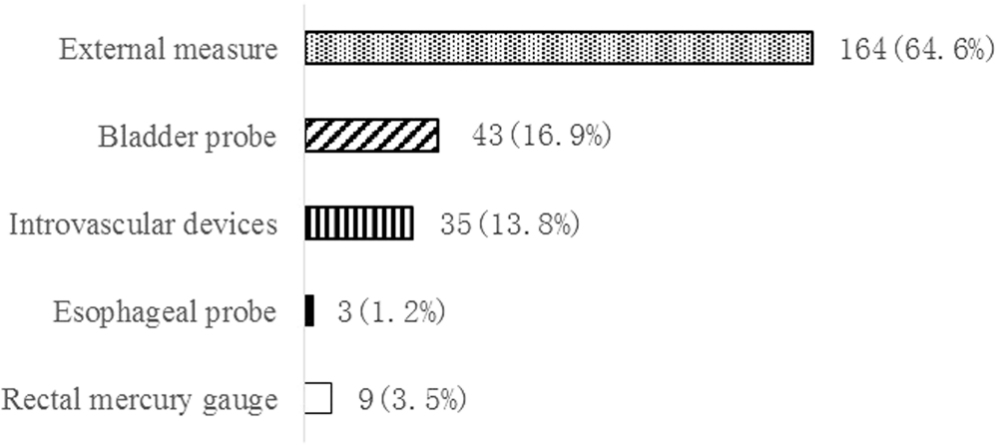
Sites of temperature monitoring during TTM in CA patients (n = 201 respondents; 254 answers expressed as percentage of response). External measure: skin measurements; Intravascular devices: Swan-Ganz catheter or PICCO measurements. TTM: target temperature management; CA: Cardiac arrest.

**Figure 3:**
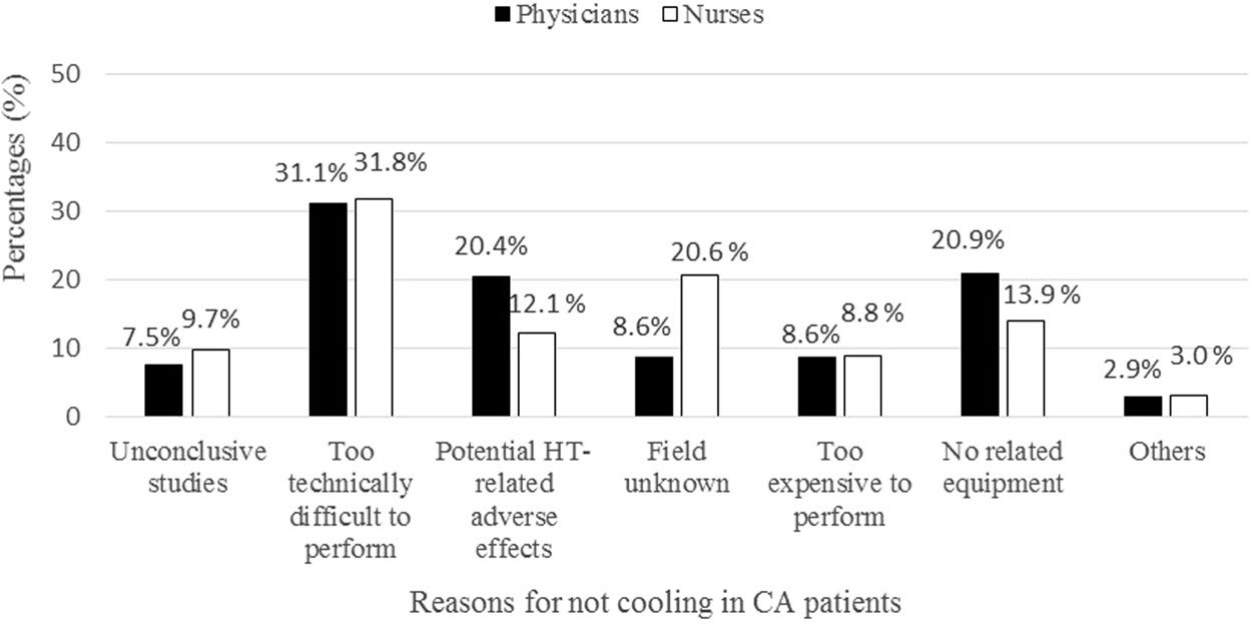
Reasons why therapeutic hypothermia is not considered in unconscious cardiac arrest patients (Physicians: n = 614 respondents; 1119 answers expressed as percentage of responses; Nurses: n = 695 respondents; 1293 answers expressed as percentage of responses). CA: cardiac arrest.

Temperature monitoring during TTM in CA patients was mainly performed using external measure as reported by 81.6% of respondents, and/or bladder probe by 21.4% of respondents (Fig. 2). TTM in CA patients was declared only by 14.5% of physicians and 6.7% of the nurses. The overall duration of TTM was mainly applied 12 h∼24 h by 36.8% of physicians, and 24 h∼48 h by 32.6%. Only 7.8% of physicians and 5.7% of the nurses had implemented TH for unconscious CA patients in practice. The top three reasons for nonuse of TH among physicians were technical difficulty of cooling (31.1%), lack of TH-related equipment (20.9%) and being afraid of potential TH-related adverse effect (20.4%) (Fig. 3).

Indications of TH implementation in CA patients are described in Table 4. TH was reported to be induced and/or maintained using cold intravenous fluid infusion and/or external water blanket more frequently, whereas external advanced surface cooling gel pads and intravascular cooling were rarely used. During the TH period, patients were reported to always receive sedatives only by 21.7% of respondents (midazolam 70.9% and/or propofol 54.7%), analgesics by 4.8% (morphine 54.3% and fentanyl 42.9%), and neuromuscular blockers by 15.8% (vecuronium 68.0% and rocuronium 16%).

**Table 4:** The TTM practice of physicians in comatose adult patients with CA after ROSC (number of respondents: n).

	Physicians
Indications for TH	
Out-of-hospital CA from initial shockable rhythm^a^	
Always	18/34(47.1)
Frequently	10/34(29.4)
Sometimes	8/34(23.5)
Never	0/34(0.0)
Do not know	0/34(0.0)
Out-of-hospital CA from initial non-shockable rhythm^b^	
Always	18/36(50.0)
Frequently	8/36(22.2)
Sometimes	8/36(22.2)
Never	2/36(5.6)
In-hospital CA from initial shockable rhythm^a^	
Always	18/36(50.0)
Frequently	6/36(16.7)
Sometimes	12/36(33.3)
Never	0/36(0.0)
Do not know	0/36(0.0)
In-hospital CA from initial non-shockable rhythm^b^	
Always	16/36(44.4)
Frequently	8/36(22.2)
Sometimes	10/36(27.8)
Never	0/36
Do not know	2/36(5.6)
Cooling methods to induce TH	
Cold intravenous fluid infusion	
Always	9/66(13.6)
Frequently	24/66(36.4)
Sometimes	24/66(36.4)
Never	9/66(13.6)
External water blanket or ice packs	
Always	21/68(30.9)
Frequently	29/68(42.6)
Sometimes	14/68(20.6)
Never	4/68(5.9)
External adhesive cooling pads	
Always	6/53(11.3)
Frequently	9/53(17.0)
Sometimes	14/53(26.4)
Never	16/53(30.2)
Do not know	8/53(15.1)
Intravascular devices	
Always	7/67(10.4)
Frequently	10/67 (14.9)
Sometimes	20/67 (29.9)
Never	22/67 (32.8)
Do not know	8/67 (11.9)
Cooling methods to maintain TH	
External water blanket	
Always	16/60(26.7)
Frequently	15/60(25.0)
Sometimes	13/60(21.7)
Never	14/60(23.3)
Do not know	2/60(3.3)
External adhesive cooling pads	
Always	4/52(7.7)
Frequently	7/52(13.5)
Sometimes	8/52(15.4)
Never	26/52(50.0)
Do not know	7/52(13.5)
Intravascular device	
Always	3/66(4.5)
Frequently	5/66 (7.6)
Sometimes	13/66(19.7)
Never	35/66(53.0)
Do not know	10/66(15.1)
Durations of the overall TTM	
<12 h	2/114(1.8)
≥12 h ≤24 h	42/114(36.8)
>24 h ≤48 h	37/114(32.5)
>48 h ≤72 h	16/114(14.0)
>72 h	13/114(11.4)
Do not know	4/114(3.5)

Results are expressed as n (%); TH: Therapeutic hypothermia; ^a^Ventricular fibrillation/pulseless ventricular tachycardia; ^b^Asystole/pulseless electrical activity.

### Changes of TTM in the future practice after publication of 2015 AHA guidelines for CPR

**Figure 4:**
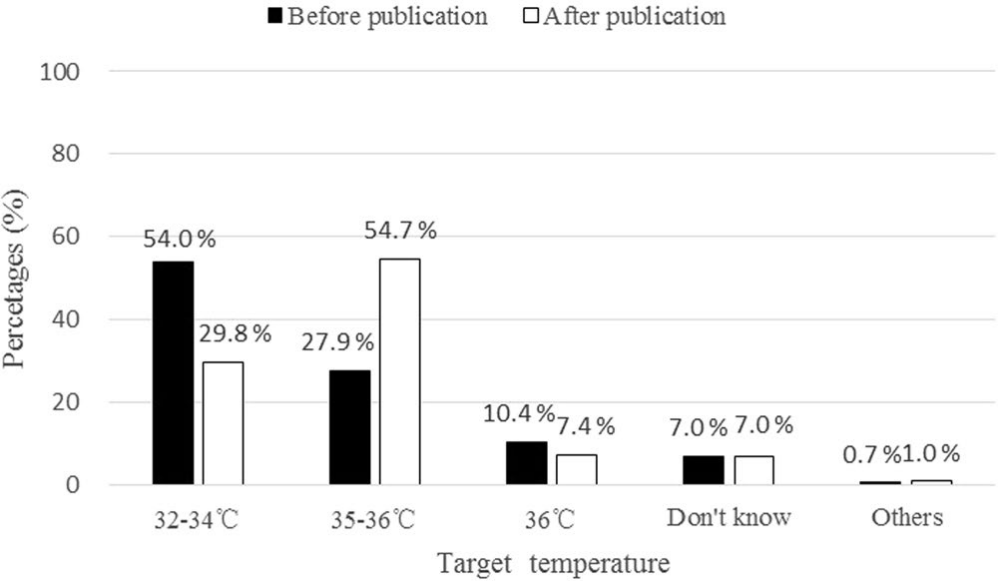
Distribution of target temperature before and after publication of 2015 AHA resuscitation guidelines (n = 298 respondents, expressed as percentage).

Among respondents who were familiar with TTM and answered the question whether they would change target temperature in future practice in CA patients after 2015 guidelines publication, 31.6% (59/187) of physicians and 38.6% (49/127) of nurses declared no modification, whereas 57.2% (107/187) of physicians and 42.5% (54/127) of nurses declared that they would change target temperature in the future TTM practice. The temperature targets preferred by respondents before and after publication of 2015 guidelines were depicted in Fig. 4. Respondents were more likely to choose 35∼36 °C-TTM (54.7%) after guidelines publication, as compared to that before guidelines publication they preferred 32∼34 °C-TTM (54.0%).

## Discussion

### Poor awareness and utilization of TTM

Our survey revealed that more than 4/5 healthcare providers of EDs or ICUs were not familiar with TTM in CA patients recommended by guidelines.TTM in CA patients was declared by 14.5% of physicians and 6.7% of the nurses. Only 4 of 64 departments, 7.8% of physicians and 5.7% of the nurses had implemented TH for CA patients in practice.

Since the two main studies published in 2002^5,6^, TH began to be used for CA patients. In the early stage, the rates of TH implementation were relatively low ranging from 13 to 27% with variations according to countries^7,14–16^. Widespread TH implementation seemed to occur after publication of 2005 resuscitation guidelines^17^. Two consecutive surveys performed in Poland found that the number of ICUs using TH increased threefold in the5-year period of 2005–2010^7,8^. Similarly, TH use increased from 28.4% to 85.6% between 2006 and 2010 in the United Kingdom^18,19^. Finally, Wils and coworkers reported up to 98.7% adherence to recommendations regarding TTM in Netherlands. Interestingly, 25% of ICUs had adapted their TTM protocol to a target temperature of 36 °C only several months after publication of TTM trial. It was a surprisingly rapid adoption of TTM practices compared to introduction of TH in the early 2000s^20^. Maybe TTM in CA patients would be more popular after 2015 resuscitation guidelines. But it was not until 2012 that TH was first used for CA patients in China. And to date only a few healthcare providers knew the concept of TTM and fewer of them had implemented it in practice. This was particularly prominent in nurses. It did reveal that healthcare providers in EDs or ICUs were failing to keep up with clinical developments and there was a huge gap between the TTM practice in China and that recommended by guidelines. In previous studies, providers have cited technical difficulties, a lack of financial or personnel resources to initiate TH^14,21^. Similarly in our study, one major impediment was that they felt too technically difficult to perform. The following two reasons for physicians were lack of TH-related equipment and being afraid of potential TH-related adverse effect. But nurses cited unknown field as the second reason for not cooling, reflecting the need for education to promote TTM awareness.

### Cooling methods

Although more than 50% of the physicians believed that intravascular cooling was better in temperature control and improvement of outcomes in CA patients, external cooling was the most common method to induce and maintain TH in practice. Similarly, other surveys observed that surface cooling were mostly used in ICUs, usually reaching about 50–60%^18,22^. Mainly because surface cooling was easy to use and could be started immediately without invasive procedure^23^. The available data on safety and efficacy of different cooling technologies is limited. Most published studies were small, often retrospective and/or had evaluated only a single cooling device or method^24^. Until 2014 a prospective multicenter RCT found that compared to external cooling, intravascular methods provided a target temperature faster, smaller temperature fluctuations in the maintenance phase and reduced nursing workload^25^. Rosman and his colleagues had got the similar conclusions later^26^. But none had found statistically significant differences in patient outcomes between different cooling methods^25–27^.

### TTM modifications after publication of 2015 AHA guidelines for CPR

Since the publication of 2015 AHA resuscitation guidelines^3^, 57.2% of physicians and 42.5% of nurses declared a target temperature’s change in future practice. Now the optimal target temperature was still unknown. A large RCT published in 2013 found no difference between strict temperature control at 33.0 °C compared to 36 °C^28^. Although the conclusions of this study have been criticized for problems such as potential selection bias, rapid rewarming, and other issues^29^. It did have an impact on guidelines and clinical practice. An international survey found that 37% of ICUs in 11 countries declared a practical target temperature change^30^. 19.2~40.4% of ICUs had adopted non 32~34 °C as new target temperature^20,30,31^. To the best of our knowledge, this is the first survey about TTM modifications after publication of 2015 guidelines.

The guidelines did have impact on attitude in choosing target temperature for TTM. The new target temperature 35 °C∼36 °C and 36 °C were preferred by 54.7% and 7.4% of the respondents respectively after guidelines publication, as compared to that 32 °C∼34 °C was preferred by 54.0% of respondents before publication. Similarly as observed in other surveys, clinicians who changed target temperature were more likely to choose a TTM set around 36°C^30,32^. But Bray and coworkers found that change from a post-arrest temperature target of 33 °C to 36 °C might lead to low compliance with target temperature and higher rates of fever which was associated with poorer patient outcomes^32^. Casamento *et al*. also reported high proportions of temperatures above target and fever (≥38 °C) across the first 24 h of ICU stay on the 36 °C protocol^33^. Mainly because shivering was usually more active at temperatures close to the normal range than at temperatures that are several degrees below normal^23,34^. Maybe it was more difficult to maintain a target temperature at 36 °C.

### Improving utilization of TTM

#### In awareness level

Our study revealed that many healthcare professionals were not familiar with TTM. This lack of knowledge might lead to a negative attitude towards TTM implementation. Such barriers were also primarily identified by Toma *et al*.^35^. Education to the physicians and nurses in EDs and ICUs, especially the chief of departments, are crucial to increase the rates of TTM utilization which had been proven effective by Morrison *et al*.^36^.

#### In practice level

The major barrier to cooling was too technically difficult to perform as surveyed in our study. It was similarly reported in previous studies^7,14–16,37^. The solution may lie in publication of standardized cooling protocols and development of regional post cardiac-arrest care system. Although TH has been recommended by resuscitation guidelines^3,10^, few specific consensus protocols exist to detail how to best implement this complex therapy. The development and publication of standardized cooling protocols will likely help physicians to better utilize this challenging treatment. Furthermore we had established regional post cardiac-arrest TTM care system led by Peking University third hospital in Beijing. The system consisted of 7 level III hospitals. Providers at dedicated TTM centers are available for any protocol or equipment related questions. But the effect needs further evaluation. In addition promoting insurance cover of TTM costs may be another effective method to improve its use in China.

It was very difficult to conduct a randomized observational survey. Convenience sampling was used in our study in order to get a high response rate. Despite surveyed hospitals were geographically well distributed in this study, it did has disadvantages of convenience sampling. The survey may have been subject to bias ascertainment as level III hospitals were over-represented, making it likely that the degree of TTM awareness and use reflected in our survey is an optimistic perspective. The true proportion may be lower than that reported in this study.

## Conclusions

TTM for CA patients had not been well understood by healthcare providers in EDs or ICUs and was still in the early stage in China. There was a huge gap between TTM practice in China and that recommended by guidelines. Interventions are needed to promote knowledge awareness and translations of guidelines into clinical practice. The publications of 2015 resuscitation guidelines did have impact on attitude in choosing target temperature for TTM. Healthcare providers were more likely to choose 35∼36 °C-TTM after guidelines publication, as compared to that before publication they preferred 32∼34 °C-TTM.

### Availability of data and material

The datasets used for this study are available from the corresponding author on reasonable request.
